# Capsular lesions with glenohumeral ligament injuries in patients with primary shoulder dislocation: magnetic resonance imaging and magnetic resonance arthrography evaluation

**DOI:** 10.1111/j.1600-0838.2010.01282.x

**Published:** 2011-12

**Authors:** S Liavaag, M G Stiris, S Svenningsen, M Enger, A H Pripp, J I Brox

**Affiliations:** 1 Department of Orthopaedic Surgery, Sørlandet HospitalArendal, Norway; 2 Department of Diagnostic Radiology, Oslo University HospitalOslo, Norway; 3 Orthopaedic Center, Skadelegevakten, Oslo University Hospital and Medical School, Ullevaal, University of OsloOslo, Norway; 4 Biostatistics and Epidemiology Unit, Oslo University HospitalRikshospitalet, Oslo, Norway; 5 Department of Orthopaedic Surgery, Oslo University HospitalRikshospitalet, Oslo, Norway

**Keywords:** Shoulder dislocation, capsular lesion, humeral avulsion of the glenohumeral ligament (HAGL), PHAGL, MRI and MR-arthrography

## Abstract

The glenohumeral ligaments are important structures for the stability of the shoulder. They are integrated parts of the capsule and are at risk to be injured in a traumatic shoulder dislocation. The aim was to examine the prevalence of capsular ligament lesions in the acute phase and at minimum 3 weeks' follow-up after first-time traumatic shoulder dislocation. Forty-two patients aged 16–40 years were included. All patients underwent computed tomography and magnetic resonance imaging (MRI) scans shortly after the injury and MR-arthrography (MRA) at follow-up. The median time from dislocation to MRI was 7 (range 2–14) days and to MRA 30 (range 21–54) days. We found capsular ligament lesions in 22 patients (52.4%) in the acute stage and in five patients (11.9%) at follow up. Nine patients (21.4%) had a humeral avulsion of the anterior glenohumeral ligament (HAGL lesion) on MRI. Three patients (7.1%) had this lesion at follow-up. The rate of HAGL lesions in the acute stage was higher than reported previously, but the prevalence at follow-up was in keeping with earlier published studies.

Avulsion of the labrum and the attached inferior glenohumeral ligament (IGHL) from the anteroinferior glenoid rim, described as the Bankart lesion, has been regarded as the essential lesion of anterior traumatic dislocations of the shoulder. However, other factors also contribute to recurrent instability. Turkel showed the importance of the glenohumeral ligaments for the stability of the glenohumeral joint (Turkel et al., 1981). Plastic deformation of the glenohumeral ligaments probably occurs at the first dislocation and progresses with recurrent dislocations (Bigliani et al., 1992; Urayama et al., 2001).

Capsular avulsion from the humerus in patients with shoulder dislocation was first described byNicola (1942). The lesion was later described and defined by Wolf et al. (1995) as the humeral avulsion of the anterior glenohumeral ligament (HAGL) lesion. Originally, the term HAGL lesion has been used for lesions of the anterior glenohumeral ligaments, but later, posterior or reversed HAGL (PHAGL or RHAGL) have been used for lesions of the humeral insertion of the posterior inferior humeral ligaments (Castagna et al., 2007). In 2007, Bui-Mansfield suggested a classification system with six different HAGL lesions (Bui-Mansfield et al., 2007).

Bigliani tested the IGHL for failure (Bigliani et al., 1992). The ligament failed at the glenoid insertion in 40%, within the substance of the ligament in 35% and at the humeral insertion site in 25%. The prevalence of IGHL tears in patients with first-time shoulder dislocation is, to our knowledge, unknown.

The HAGL lesion has been reported to occur in 7.5–9.4% of patients undergoing arthroscopic surgery for anterior instability and in 1.0–9.0% of patients with recurrent instability (Bokor et al., 1999; Bui-Mansfield et al., 2002). In one study with arthroscopic control after traumatic anterior dislocation, the rate of HAGL lesion was reported to be as low as 1.6% (1/63) (Taylor & Arciero, 1997).

The aim of the present study was to examine the prevalence of glenohumeral ligament lesions evaluated by magnetic resonance imaging (MRI) in the acute phase after primary anterior shoulder dislocation and compare it with the prevalence of capsular ligament lesions in the same shoulders examined with MR-arthrography (MRA) at minimum 3 weeks and maximum 3 months of follow-up.

## Material and methods

The study was approved by our institutional study board, the Regional Medical Ethics Committee and the Norwegian Social Sciences Data Services.

### Patients

Patients were recruited from Oslo University Hospital, Ullevaal and Akershus University Hospital. The inclusion criteria were as follows: patients aged 16–40 years with primary traumatic anterior glenohumeral dislocation successfully reduced and documented by conventional radiographic examination before and after reduction. The exclusion criteria were: (1) Previous shoulder dislocation. (2) Fracture of the glenoid with a large bony defect of the glenoid rim (including >20% of the length of the glenoid rim), or a bony glenoid defect involving more than 1/3 of the diameter of the glenoid fossa at the same level (Burkhart & De Beer, 2000). (3) Fracture of the greater tuberosity of the humerus, with malalignment after reduction. (4) Nerve damage related to dislocation or reduction (plexus damage or damage of the axillary nerve). (5) The patient was not willing or able to carry out the investigation. (6) The patients were excluded if the MRI was not conducted within 14 days or if the MRA was conducted earlier than 3 weeks or later than 3 months after the injury. All patients were treated with immobilization for 3 weeks in either internal or external rotation, 21 patients in internal and 21 patients in external rotation.

### Imaging

Imaging was conducted at the Department of Radiology at Oslo University Hospital-Aker. One specialized radiologist described the images and filled in a standardized questionnaire. The radiologist has long experience in MRI of the shoulder and particular interest in musculo-skeletal radiology. MRI was conducted in the acute phase within 14 days after the dislocation and by MRA at minimum 3 weeks and maximum 3 months of follow-up.

MRA is probably the most reliable diagnostic imaging technique for detecting labroligamentous lesions (Waldt et al., 2005). The presence of joint effusion or intraarticular injection of contrast fluid is necessary to detect HAGL lesions on MR images (Stoller, 1997; Bui-Mansfield et al., 2002). Taylor and Arciero reported hemarthrosis in all of the 63 patients who underwent arthroscopy within 10 days and Wintzell et al. (1996) reported that 97 % of the patients examined with MRI within 10 days after primary traumatic dislocation had joint effusion (Taylor & Arciero, 1997). Based on these reports, we assumed that during the first 2 weeks after a shoulder dislocation, there would still be blood and effusion in the joint cavity acting as a contrast fluid.

The MRI and MRA examinations were performed on a 1.5 T scanner. The following sequences were obtained in the MR evaluation: oblique coronal PD/T2-weighted sequence (FoV 200 × 150, matrix 192 × 192, TR: 3070, TE 13, TE 79) and STIR (T1 inversion recovery, FoV 200 × 159.4, matrix 157 × 256, TR 5000, TE 29), axial PD fs (fat saturation) (FoV 175 × 175, matrix 248 × 266, TR 3000, TE 13) and sagittal oblique T2 (FoV 190 × 190, matrix 192 × 192, TR 3500, TE 88). A 4 mm slice thickness with a 0.8 mm gap and an acquisition of 1 were used in all sequences.

Before the MRA examination, an arthrogram was performed fluoroscopically under sterile conditions. Afterwards, the images were obtained, using the following sequences: Oblique coronal PD/T2 (FoV 200 × 150, matrix 192 × 256, TR 3000, TE 15, TE 93) and T1 fs (FoV 175 × 131.3, matrix 146 × 256, TR 490, TE12), axial T1 fs (FoV 160 × 160, matrix 256 × 256, TR490, TE 12), oblique sagittal T1 (FoV 160 × 1660, matrix 256 × 256, TR 558, TE 12) and abduction external rotation T1 (FoV 160 × 160, matrix 256 × 256, TR 551, TE 12). The slice thickness was 4 mm with a gap of 0.8 mm with an acquisition of 2. A multidetector scanner was used for computed tomography (CT) scans. Both bone and soft tissue windows were performed in the CT algorithm. An axial volume (64 × 0.6 mm) was performed through the acromioclavicular and glenohumeral joints. Then, an oblique coronal and an oblique sagittal projection were reconstructed directly from the raw data. The MRI, MRA and CT examinations were sent to the Picture Archive and Communication System archive for evaluation.

### Classifications

**Fig. 2:**
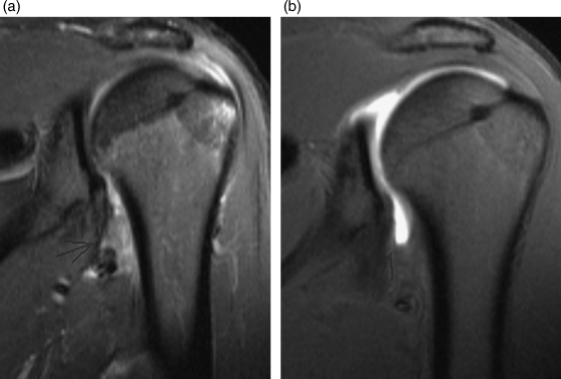
A posterior anterior glenohumeral ligament (HAGL) lesion disappearing after 31 days. The figure shows the images from the shoulder of a 29-year-old male after primary traumatic anterior dislocation of the left shoulder. (a) Oblique coronal STIR (3000/37) 8 days after dislocation of the left shoulder reveals a posterior HAGL lesion. (b) Oblique coronal T1 fat sat (600/11) with intraarticular contrast medium obtained 31 days after the dislocation demonstrates full recovery.

The lesions were defined according to the definitions used in the routine radiographic descriptions at the clinic. HAGL lesions were registered when the attachment of the IGHL was avulsed from the humeral attachment and inferior extravasations of fluid (blood, effusion or contrast liquid) with the typical “J” sign in the right shoulders and a reversed “J” sign in the left shoulders on coronal oblique views (Bui-Mansfield et al., 2002; Carlson, 2004). Lesions without the typical “J” or reversed “J” were classified as HAGL lesions if the IGHL was clearly visible and if there was a defined inhomogeneity or frank disruption from the humeral attachment and extravasations of fluid. See Fig. 2. PHAGL was defined as a definite avulsion of the glenohumeral ligament at the posterior aspect of the capsule. Lesions were defined as IGHL tears if a definite defect in the ligament substance was detected. In addition to the descriptive comments in the radiological journal, a standard questionnaire was used to register CT, MRI and MRA findings.

The Bankart lesion was defined as an avulsion of the anterior capsulolabral complex inferior to the equator of the glenoid but although the Bankart lesion affects the glenohumeral ligament at the attachment on the glenoid, we categorize it as a labral lesion in the present study. Anterior labral-periostal sleeve avulsion (ALPSA) lesion and Superior labrum anterior to posterior lesion (SLAP) were defined according to standard definitions (Snyder et al., 1990; Neviaser, 1993).

Cuff tears were described as full thickness or partial tears, describing the number of tendons affected and the degree of retraction.

All patients were also examined with CT to detect and describe the bony lesions. Hill–Sachs lesions were graded as small (mild), medium (moderate) and large (severe) according to the definition of Calandra et al. (1989).

### Statistical analysis

Descriptive statistics are presented in the text and in Table 1. The median (range), mean values (SD) and percentages are given. Differences between groups were analyzed using the Mann–Whitney or the chi-square test as appropriate. Differences in the frequency of HAGL lesion and IGH tear on MRI and MRA were analyzed using Mc Nemars test. Gamma for trend was used to test the association between HAGL and Hill–Sachs lesions in the acute stage.

**Table 1:** Capsular lesions with affection of the glenohumeral ligaments or labral lesions in patients with primary traumatic anterior shoulder dislocation evaluated with MRI at median 7 days after the dislocation and MRarthrography after median 30 days

Lesions	MRI	MRA	*P* value*

Capsular			
HAGL	9	3	0.03
Ligament lesions			
PHAGL	13(8)†	2(2)†	0.001
IGHL tear	14(6)‡	4(2)‡	0.002
Bankart lesion	34	21	0.000
Labral lesions			
ALPSA	0	5§	
SLAP	7	7	1.00

Number of patients are reported (*n*=42).

* Mc Nemars test.

† Combined HAGL and PHAGL.

‡ Tears in the ligament combined with HAGL or PHAGL.

§ Converted from Bankart on MRI.

At MRI 14 of the lesions were combined lesions and in total there were 22 patients with capsular lesions with injury of the glenohumeral ligament on MRI. At MRA 4 of the lesions were combined lesions and in total there were five patients with injuries of the capsule affecting the glenohumeral ligaments.

## Results

### Patient characteristics and time from injury to imaging

Forty-two patients were included. The mean age of the patients included was 26.7 years (SD 1.1) and the range of age was from 16 to 39 years. There were 33 men and nine women.

The median time from dislocation to MRI was 7 (range 2–14) days and the median time to MRA was 30 (range 21–54) days. None of the patients were excluded because of CT findings. All patients still had blood and effusion in the joint cavity acting as contrast fluid when the MRI examination was performed. No technical problems were reported with the imaging because of lack of contrast fluid. A quantification of the amount of fluid in the joint was not performed.

### Glenohumeral ligament injuries in the acute stage

In total, we found capsular lesions with affection of the glenohumeral ligaments in 22 patients (52.4%). Nine patients (21.4%) had a HAGL lesion, an isolated HAGL lesion was described in one patient and in eight cases the HAGL lesion was combined with a posterior HAGL. Five patients had only a posterior HAGL on MRI. In total, a HAGL lesion or a PHAGL were present in 14 (33.3%) of the patients on MRI. IGHL tears were also present in 14 of the patients but for six patients (14.3%) the IGHL tear was combined with a HAGL or a PHAGL lesion (Table 1).

### Glenohumeral ligament injuries at follow-up

Five patients (11.9%) still had a capsular lesion with affection of the glenohumeral ligaments at follow-up. Six of the initially nine HAGL lesions on MRI (66.7%) were not detectable at follow-up and only three patients (7.1%) had a persistent HAGL lesion, one isolated HAGL lesion and two combined with a posterior HAGL lesion. None of the HAGL lesions visible on MRA were undetected on the initial MRI. All three patients with persistent HAGL lesions were women.

**Fig. 1:**
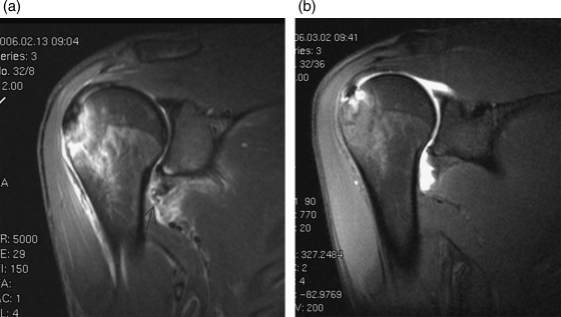
An anterior humeral avulsion of the anterior glenohumeral ligament (HAGL) lesion disappearing after 3 weeks. The figure shows the images from the shoulder of a 27-year-old male, after primary traumatic anterior dislocation of the right shoulder. (a) Oblique coronal STIR (3000/37) 6 days after dislocation of the right shoulder demonstrates an anterior HAGL lesion (arrow). (b) Oblique coronal T1 fat sat (600/11) with intraarticular contrast medium 24 days after the dislocation reveals full recovery.

Four patients (9.5%) had an IGHL tear at follow-up but for two of the patients these lesions were described as a combination of an IGHL tear combined with HAGL or PHAGL. Figs 1 and 2 show examples of lesions discovered in the acute stage that had disappeared at follow-up.

### Labral lesions, bony lesions and tendon injuries

We found a Bankart lesion in 34 (81%) patients on MRI; 21 were persistent on MRA. We did not find any patients with an ALPSA lesion on the initial MRI, but in five patients, the Bankart lesion was transformed from a Bankart lesion on MRI to a periostal sleeve avulsion (ALPSA) on MRA. We found seven SLAP lesions on MRI, and they were all combined with Bankart lesions and did not disappear on MRA.

Thirty-nine patients (92.9%) had a Hill–Sachs lesion on CT. Six patients (14.3%) had a fracture of the greater tuberosity. All fractures of the greater tuberosity were small non-displaced fractures. The mean age for the six patients with fracture of the greater tuberosity was 26.8 years and for those without fracture the mean age was 26.7 years. One patient had a small partial infraspinatus tear visible on MRI that had disappeared at follow-up.

### Comparison of findings

We found significantly (*P*=0.03) fewer HAGL lesions at follow-up compared with the acute stage. Significant differences were also found separately for anterior and posterior HAGL, respectively (Table 1). There was no significant difference in the prevalence of capsular ligament lesions between males and females. We did not find any correlation between age and type of lesions either for capsular ligament lesions, labral lesions, Hill–Sachs lesion or fracture of the greater tuberosity. The prevalence of HAGL and PHAGL lesion was inversely correlated with the size of the Hill–Sachs lesion (Table 2). We found these lesions in nine of the 11 patients with small or absent Hill–Sachs lesions compared with five of the 31 patients with moderate or large Hill–Sachs lesions, *P*<0.001 (Table 2). The difference in the number of Bankart lesions found on MRI and MRA was also significant, *P*=0.000.

**Table 2:** Grading of the Hill–Sachs lesions combined with a HAGL or a PHAGL lesion

	CT Grading of Hill–Sachs lesions				
	
	No lesion	Small	Moderate	Large	Total
MRI (HAGL or/and PHAGL) Present/Total	3/3	6/8	2/9	3/22	14/42
MRA (HAGL or/and PHAGL) Present/Total	1/3	2/8	0/9	0/22	3/42

Number of patients are given (*n*=42).

*P*<0.001 (Gamma for trend of MRI lesions in the acute stage).

At MRA all three remaining HAGL lesions were among patients with only small or absent Hill–Sachs lesions.

## Discussion

To our knowledge, this is the first study comparing the findings at MRI shortly after a primary shoulder dislocation with MRA at follow-up. We found a high prevalence of capsular ligament lesions on MRI shortly after the primary shoulder dislocation. At follow-up, the prevalence of HAGL lesions was significantly reduced and in agreement with previous studies (Wolf et al., 1995; Bokor et al., 1999; Bui-Mansfield et al., 2002). The prevalence of PHAGL and IGHL tears was also significantly reduced.

The strengths of the present study are the inclusion of a relatively large group of young patients with primary anterior traumatic shoulder dislocation documented by conventional radiographic examination before reduction, and that imaging was conducted and described in a standardized manner by a radiologist experienced in MRI of the shoulder.

The main limitation of this study is the lack of surgical correlation, but none of the patients were operated during the observation period. Another limitation is the lack of estimation of intraobserver error. In addition, the radiologist was not blinded for MRI findings when he described the MRA. The lack of surgical correlation and blinded interrater agreement assessment introduces a potential bias in the study and misinterpretation of some of the images cannot be excluded.

The prevalence of labral injury, tendon tears and Hill–Sachs lesions was in agreement with a recently published MRA-based study by Antonio et al. (2007) assessing abnormalities after first-time shoulder dislocation. All over, they found more cuff tears (27%), but only one of 34 patients younger than 30 years had a cuff tear. In our study, one patient had a partial rotator cuff tear on MRI. Further, Antonio et al. reported that 71% of the patients had a Hill–Sachs lesion, which is slightly <92.9% in the present study and 81% in the CT-based study by Griffith and colleagues in 2007 (Griffith et al., 2003). Norlin reported in his arthroscopic study concerning intraarticular pathology in acute first-time anterior shoulder dislocation that all shoulders showed Bankart and Hill–Sachs lesion (Norlin, 1993).

We found an increased risk of HAGL or PHAGL lesion among patients with small or absent Hill–Sachs lesions compared with patients with a large or moderate Hill–Sachs lesion (Table 2). We are not aware of any studies reporting similar findings and further studies are warranted to explore possible mechanisms.

Earlier studies have reported that 20% of patients with a HAGL lesion have a bony avulsion from the medial cortex of the humeral head (Bui-Mansfield et al., 2002).We did not find bony HAGL lesions.

Earlier cadaveric studies have postulated that more than a Bankart lesion alone is probably required for a dislocation to occur (Pouliart & Gagey, 2006; Pouliart & Gagey, 2008). Bui-Mansfield stated in his review article in 2002 that little has been written about the HAGL lesion in the radiological literature (Bui-Mansfield et al., 2002). Waldt et al. (2005) concluded that MRA is accurate in enabling the classification of acute and chronic anteroinferior labroligamentous injuries. Most patients in earlier studies have been examined a long time after their initial shoulder dislocation either at surgery or at a preoperative MRA. In Wolf's study, the average duration of preoperative instability was almost 3 years (Wolf et al., 1995).

Contradictory results are reported in two studies evaluating patients arthroscopically in the acute stage. Baker et al. (1990) reported capsular tears in all 45 patients who underwent arthroscopic evaluation within 10 days after acute anterior shoulder dislocation. In contrast, Taylor & Arciero (1997) found one HAGL lesion in the 63 patients examined arthroscopically within 10 days of first-time traumatic anterior dislocation.

Melvin et al. (2008) presented clinical observations of four patients with preoperative MRI diagnosis of HAGL who had no evidence of HAGL at later arthroscopy. All the patients had defects in the IGHL complex that simulated the appearance of HAGL on MRI. They concluded that the exact diagnosis of HAGL should be reserved for arthroscopy, and recommended describing MRI findings using a more general term like a defect of the IGHL complex. We agree that the lack of coincidence in the findings between MRI/MRA and arthroscopy might be a question of definitions, but both imaging methods may visualize capsular injuries in greater detail than we are able to observe during an operation. However, improved knowledge concerning the reliability and validity of findings at arthroscopy and imaging is warranted.

In a letter to the AJR editor in response to Melvin's article, Murphy et al. (2009) reported three cases of HAGL lesions detected on conventional MRI scans that were not seen on subsequent MRA scans. They hypothesized that the HAGL lesion may heal spontaneously as the shoulder capsule is a vascular structure. Neither the cited study nor the present study was designed to assess healing, but the disappearance of the lesions reported in the present study may have relevance for the treatment of patients with anterior primary shoulder dislocation. We cannot exclude that the methodological limitations may influence our results; however, our findings at MRI and MRA indicate that there are true traumatic lesions in the ligamentous part of the capsule that disappear during the first 3 months after a primary traumatic shoulder dislocation. Further studies with assessment of interobserver reliability and arthroscopic blinded interobserver agreement and arthroscopic evaluation with clinical findings are warranted.

The most likely clinical relevance of our findings is that capsular lesions discovered with MRI in an early phase after the injury are likely to disappear. Consequently, routine MRI is not indicated in the acute stage and if applied the clinical relevance of pathological findings in the capsular ligament structures should be interpreted with caution. If further diagnostic is needed we suggest MRA after a couple of months.

## Perspectives

The prevalence of HAGL lesions in the acute stage was much higher than reported previously, but the prevalence at follow-up was in keeping with earlier published studies. Despite the limitations of the present study, our findings suggest spontaneous disappearance of capsular ligamentous lesions in the shoulder after primary shoulder dislocation.
